# Drivers of Collembola assemblages along an altitudinal gradient in northeast China

**DOI:** 10.1002/ece3.8559

**Published:** 2022-02-12

**Authors:** Zhijing Xie, Xin Sun, Johannes Lux, Ting‐Wen Chen, Mikhail Potapov, Donghui Wu, Stefan Scheu

**Affiliations:** ^1^ Key Laboratory of Wetland Ecology and Environment Northeast Institute of Geography and Agroecology Chinese Academy of Sciences Changchun China; ^2^ Key Laboratory of Urban Environment and Health Institute of Urban Environment Chinese Academy of Sciences Xiamen China; ^3^ University of Chinese Academy of Sciences Beijing China; ^4^ 9375 J.F. Blumenbach Institute of Zoology and Anthropology University of Göttingen Göttingen Germany; ^5^ Biology Centre of the Czech Academy of Sciences Institute of Soil Biology České Budějovice Czech Republic; ^6^ 386213 Moscow State Pedagogical University Moscow Russia; ^7^ Key Laboratory of Vegetation Ecology Ministry of Education Northeast Normal University Changchun China; ^8^ Jilin Provincial Key Laboratory of Animal Resource Conservation and Utilization Northeast Normal University Changchun China; ^9^ 9375 Centre of Biodiversity and Sustainable Land Use University of Göttingen Göttingen Germany

**Keywords:** climatic and environmental factors, community, elevation, forest, soil animal, springtails

## Abstract

Altitudinal changes in the diversity of plants and animals have been well documented; however, soil animals received little attention in this context and it is unclear whether their diversity follows general altitudinal distribution patterns. Changbai Mountain is one of few well‐conserved mountain regions comprising natural ecosystems on the Eurasian continent. Here, we present a comprehensive analysis of the diversity and community composition of Collembola along ten altitudinal sites representing five vegetation types from forest to alpine tundra. Among 7834 Collembola individuals, 84 morphospecies were identified. Species richness varied marginally significant with altitude and generally followed a unimodal relationship with altitude. By contrast, the density of Collembola did not change in a consistent way with altitude. Collembola communities changed gradually with altitude, with local habitat‐related factors (soil and litter carbon‐to‐nitrogen ratio, litter carbon content, and soil pH) and climatic variables (precipitation seasonality) identified as major drivers of changes in Collembola community composition. Notably, local habitat‐related factors explained more variation in Collembola assemblages than climatic variables. The results suggest that local habitat‐related factors including precipitation and temperature are the main drivers of changes in Collembola communities with altitude. Specifically, soil and litter carbon‐to‐nitrogen ratio correlated positively with Collembola communities at high altitudes, whereas soil pH correlated positively at low altitudes. This documents that altitudinal gradients provide unique opportunities for identifying factors driving the community composition of not only above‐ but also belowground invertebrates.

## Graphical abstract

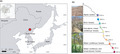

## 1 INTRODUCTION

How patterns of species richness, community structure, and their underlying drivers vary along environmental gradients is a key question in ecology. Mountain regions are hot spots for biodiversity, covering a wide range of abiotic factors that vary with altitude (Antonelli et al., 2018). Mountains therefore provide a unique opportunity to study changes in density and community composition of plants and animals along these environmental gradients (Brehm et al., 2013; Guo et al., 2013; Li et al., 2021; Traunspurger et al., 2017). Plants and animals have to adapt to environmental changes, and altitudinal variation in community composition reflects these adaptations. Since temperature and precipitation are known to change with altitude, altitudinal transects may provide information about the response of plant and animal communities to global climate change (Pauli et al., 2012), and contribute to a comprehensive understanding of climate change influences on ecosystems (Grytnes & McCain, 2007; Lomolino, 2001; Malhi et al., 2010; Rahbek, 2005).

Plants and aboveground animals (such as mammals, birds, and insects; McCain & Grytnes, 2010) show either monotonically decreasing or hump‐shaped richness patterns with altitude, potentially driven by local habitat‐related, climatic, spatial, historical or biotic factors (Bai et al., 2011; Hodkinson, 2005; Hoiss et al., 2012; Kessler, 2001). However, we know little about the influence of altitudinal gradients on soil animals, which in part is due to the paucity of taxonomical knowledge. This is particularly true for soil invertebrates outside Europe and North America (Brehm et al., 2003; Guo et al., 2013; McCain & Grytnes, 2010; Wu & Lei, 2013). Some recent studies have investigated changes in the community structure of soil invertebrates across altitudinal gradients in temperate and tropical ecosystems (Scheu et al., 2008; Traunspurger et al., 2017; Xu et al., 2015). Density and species diversity of oribatid mites have been found to decline with increasing altitude (Illig et al., 2010), while testate amoebae showed a hump‐shaped pattern peaking at intermediate altitude (Krashevska et al., 2007). In Collembola, one of the most abundant groups of soil microarthropods, the reported patterns are inconsistent.

Collembola are among the most widespread arthropods occurring in almost all terrestrial ecosystems and play important roles in ecosystem processes, such as carbon and nitrogen cycling, soil microstructure formation, and plant litter decomposition (Deharveng, 2004; Hopkin, 1997). Density and diversity of Collembola vary with environmental factors and plant community composition (Eisenhauer et al., 2011; Johnson et al., 2005; Sabais et al., 2012), likely resulting in distinct patterns along altitudinal gradients. Loranger et al. (2001) found a hump‐shaped pattern of Collembola species richness with altitude in temperate forests, driven by local habitat‐related factors, particularly soil pH. By contrast, Illig et al. (2010) observed decreasing Collembola density with increasing altitude, probably due to low resource quality at high altitude tropical montane rainforests. In addition to local habitat‐related factors, Collembola are affected by climatic variables that change with altitude, such as temperature and precipitation (Bokhorst et al., 2018; García‐Gómez et al., 2009). Increasing temperature may reduce the life span of Collembola by affecting their physiological and reproductive activities (Mertens et al., 1983; Snider & Butcher, 1972). Moreover, Ferguson and Joly (2002) uncovered a positive correlation between Collembola density and precipitation, as well as experimental water supplementation. Early studies mainly considered local habitat‐related factors and climatic variables separately, whereas recent studies combined climatic and local habitat‐related factors; for example, the assembly of nematodes was shown to be more closely related to climatic than to local habitat‐related variables (Li et al., 2020). To improve our understanding of the underlying mechanisms shaping distribution patterns in Collembola, studies including both local and climatic factors along altitudinal gradients are needed.

To fill this knowledge gap, we investigated the distribution patterns of Collembola and their main drivers in temperate forests across an altitudinal gradient of Changbai Mountain in northeast China. Changbai Mountain is one of few well‐conserved natural ecosystems (He et al., 2005) on the Eurasian continent (Xu et al., 2004). Detailed work on community assembly has been conducted at Changbai Mountain, including altitudinal changes in forest vegetation (Bai et al., 2011; Wang et al., 2019), soil microorganisms (Shen et al., 2013, 2014), and beetles (Zou et al., 2013, 2014). Notably, neither community patterns in plants, microorganisms, and invertebrates, nor the identified drivers of community assembly were consistent across taxa. Based on the results of previous studies on variations in soil mesofauna communities with altitude, we hypothesized that (1) Collembola species richness and abundance show a hump‐shaped pattern with altitude; (2) community composition of Collembola changes gradually with altitude, that is, communities of low and high altitudes are most distinct; (3) soil pH, temperature, and precipitation are critical factors affecting Collembola community composition; (4) local habitat‐related factors explain less variation in Collembola assemblages than climatic variables, such as precipitation seasonality.

## 2 MATERIALS AND METHODS

### 2.1 Study sites

The study was conducted in the Changbai Mountain Nature Reserve (henceforth Changbai Mountain) in northeast China (41°41'–42°51'N; 127°43’–128°16'E), which was established in 1960 and contains one of the best‐protected mature temperate forests in Asia (Stone, 2006; Xue & Tisdell, 2001). Changbai Mountain is characterized by exceptionally high diversity of plants and invertebrates in the temperate zone, presumably exceeding that in Europe and North America at the same latitude (Yang & Xu, 2003; Zou et al., 2013).

Changbai Mountain is characterized by a temperate continental monsoon climate, and experiences dry windy springs, short rainy summers, and cool autumns with high frequency of fog and long cold winters. Changbai Mountain can be divided into five vertical vegetation zones: (1) mixed coniferous and broad‐leaved forest zone below 1100 m, (2) mixed coniferous forest zone between 1100 and 1500 m, (3) subalpine mixed coniferous forest zone between 1500 and 1800 m, (4) birch forest zone between 1800 and 2100 m, and (5) alpine tundra above 2100 m (Bai et al., 2011; Zou et al., 2014). Samples were taken on the northern slope of Changbai Mountain spanning from 800 to 2150 m asl.

### 2.2 Sampling procedure

**FIGURE 1:**
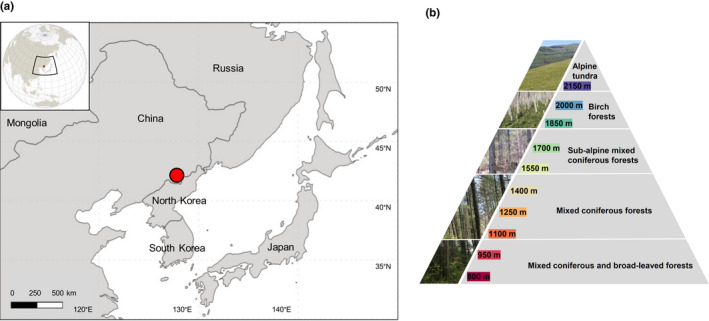
Schematic view of the sampling locations along an altitudinal gradient spanning from 800 to 2150 m at Changbai Mountain, China, modified from Xie et al. 2022

In July 2015, we collected litter and soil samples from five randomly selected plots (each measuring 10 m^2^) at each of the 10 altitudes: 800, 950, 1100, 1250, 1400, 1550, 1700, 1850, 2000, and 2150 m (Figure 1). The five plots per altitude were spaced by at least 100 m. From each plot, three random subsamples of litter and soil were collected and pooled as one litter and one soil sample. Litter samples were taken within a 100‐cm^2^ frame; soil samples were taken using 5.5‐cm‐diameter cores to a depth of 10 cm underneath the litter samples. Animals were extracted from litter and soil using Berlese funnels (diameter 20 cm, mesh size for litter 2 mm, mesh size for soil 0.84 mm) over ten days without heating, and preserved in 95% ethanol for further identification. The extraction was started as soon as possible but no longer than 24 h after collection.

### 2.3 Species identification

Collembola were separated from other soil animals and sorted into morphological species under a stereomicroscope (STEMI 508, Zeiss, Jena, Germany) based on morphological characters. At least eight individuals of each morphological species from each sample subsequently were cleaned with lactic acid, mounted in Hoyer's solution and inspected using a Zeiss Axio Scope A1 microscope. Collembola were identified to described species or morphospecies using relevant publications (Christiansen & Bellinger, 1998; Potapov, 2001; Sun et al., 2020; Xie et al., 2019). Immature specimens were sorted into morphospecies by reference to adults or subadults found in the same sample or in additional samples taken in the vicinity to support identification work. Morphospecies were classified into three ecomorphological life‐forms, that is, euedaphic (soil‐dwelling), hemiedaphic (litter‐dwelling), and epedaphic (surface‐dwelling) following Gisin (1943), Hopkin (1997), and Widenfalk et al. (2015).

### 2.4 Environmental variables

To identify potential drivers of Collembola communities and diversity along the altitudinal gradient, we used two categories of variables: local habitat‐related factors and climatic variables. Local habitat‐related factors were determined from litter or soil samples after extracting soil animals, grinded in a ball mill, and included ten variables (Table S1). Soil pH was measured after shaking a 1:5 wt/vol soil:water suspension for 30 min. Soil organic matter (SOM) was measured using an organic carbon analyzer (TOC‐V, SSM‐5000A, Japan) based on non‐dispersive infrared method. Total carbon (TC) and total nitrogen (TN) of litter and soil were measured by an elemental analyzer (vario MACRO cube, Elementar, Germany), and total phosphorus (TP) of litter and soil was determined using H_2_SO_4_‐HClO_4_ digestion. Carbon‐to‐nitrogen ratio (C/N ratio) was calculated from TC and TN for both litter and soil. The four climatic variables included in our study were mean annual temperature, mean annual precipitation, temperature seasonality, and precipitation seasonality (standard deviation ×100) retrieved from WorldClim version 2 at a 30‐s resolution (https://www.worldclim.org/; Fick & Hijmans, 2017; Table S1). Climatic data were extracted for the mean coordinates of the sampling plots from the ten altitudes using the R package “raster” (Robert 2021).

### 2.5 Statistical analysis

Abundance of species from litter and soil of the same sampling point was calculated on the basis of one square meter. Area‐based density and species richness were analyzed as count data (individuals per species per sample). The full species list and density across altitudes are given in Table S2. All analyses were performed in R software version 4.0.4 (R Core Team, 2021). Species accumulation curves were computed with the function “specaccum” in the “vegan” package (Oksanen et al., 2019) and used to inspect whether the sampling effort was adequate. Rarefaction curves were calculated for each altitude to evaluate the total sampling effort (Hsieh et al., 2016).

To test hypothesis 1, we fitted linear and (in part) quadratic models with response variables including species richness, abundance, relative abundances of different families and life‐forms, and proportional abundance in the litter layer (abundance in litter/combined abundance in litter and soil) as response variables, and altitude was fitted as explanatory variable. Pairwise differences in the response variables among altitudes were assessed using Tukey's HSD test as implemented in the package “emmeans” (Russell, 2020). To test for relationships between richness and altitude, the squared altitude was included as fixed effect. All models met the assumptions of normality of residuals and homogeneity of variance.

To test hypothesis 2, the Bray–Curtis dissimilarity matrix was calculated and non‐metric multidimensional scaling (NMDS) was used to visualize the overall differences in Collembola communities across altitudes using the “vegan” package (Oksanen et al., 2019). Only species occurring in at least three plots were included in this and following analyses, since species occurring in one or two plots reached uniformly low densities.

To test hypothesis 3, we first evaluated correlations among the environmental variables through Pearson correlation analyses using the “chart. Correlation” function in the “PerformanceAnalytics” package (Brian & Peter, 2020). Variables with correlation coefficients larger than 0.70 were considered strongly correlated and removed from further analyses (Dormann et al., 2013; for results, see Figure S6), resulting in eight factors being included in further analyses (TC and C/N ratio of soil and litter, soil pH, soil P, temperature seasonality, and precipitation seasonality). Litter species identity was not included in the analyses, as previous studies showed litter quality to be more important than litter identity in driving soil microarthropod community composition (Marian et al., 2018; Peng et al., 2022). Community data were standardized using “Hellinger” transformation and environmental data using “standardize” of the function “decostand” in the “vegan” package (Oksanen et al., 2019). Detrended correspondence analysis (DCA) recovered a length of gradient <3, indicating redundancy analysis (RDA) as appropriate approach for further analyses. RDA was run on non‐collinear factors to identify factors significantly affecting Collembola assemblages, following a forward selection model with the “ordistep” function in the “vegan” package. The Monte Carlo tests with 999 permutations were performed to evaluate overall model significance. Variation explained by the selected environmental variables was assessed by adjusted *R* ^2^ values. Scaling option was set as “species,” and the 23 species closely correlating with the first two axes were displayed.

To test hypothesis 4, we separated the variables that determined Collembola community composition selected by the procedure described above into two categories, that is, local habitat‐related factors (soil pH, litter C, soil C/N, and litter C/N) and climatic variables (precipitation seasonality). The proportion of the total variation in Collembola assemblages explained by the two categories was calculated using the sum of all canonical eigenvalues (Borcard et al., 1992) and was conducted using the “varpart” function in the “vegan” package. To evaluate the performance of the different models, permutation tests (1000 permutations, pseudo‐F statistics) were performed using the “anova.cca” function in the “vegan” package.

## 3 RESULTS

### 3.1 Species richness and density

**FIGURE 2:**
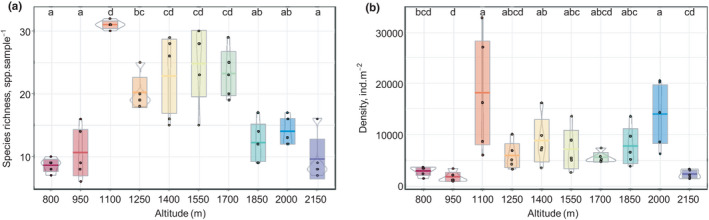
Changes in species richness (a) and density (b) of Collembola along an altitudinal gradient spanning from 800 to 2150 m at Changbai Mountain. Bars sharing the same letter do not differ significantly (*p* > .05; Tukey's HSD test) (dots = data points, bars = means, boxes = 95% confidence intervals, gray lines = density distribution)

A total of 84 Collembola species or morphospecies of 12 families were identified from a total of 7,834 individuals inspected under the stereomicroscope. Species accumulation curves became marginally asymptotic, indicating that most species present at Changbai Mountain had been sampled (Figure S1a). Rarefaction curves stabilized at about 43 and 26 species at 1400 and 1850 m, respectively, but did not stabilize at the other altitudes (Figure S1b). Average species richness varied marginally significant with altitude and generally changed in a unimodal way with altitude (*F*_1,48_ = 3.52, *p* = .067; quadratic regression analysis), but with a maximum at 1100 m (Figure 2a). This pattern was mainly due to Collembola species richness in litter, which changed significantly with altitude (*F*_1,48_ = 5.35, *p* = .025). By contrast, species richness in soil varied little with altitude with the exception of very low richness at 950 m (Figures S2a,c). Generally, more species occurred in the litter layer than in the soil, with the exception at 800 and 2150 m (Figure S3a). The density of Collembola did not change in a consistent way with altitude (Figure 2b and Figure S2b,d). Generally, the density in litter exceeded that in soil across all altitudes except at 800 m and 1850 and 2150 m (Figure S3b).

### 3.2 Families, life‐forms, and species composition

**FIGURE 3:**
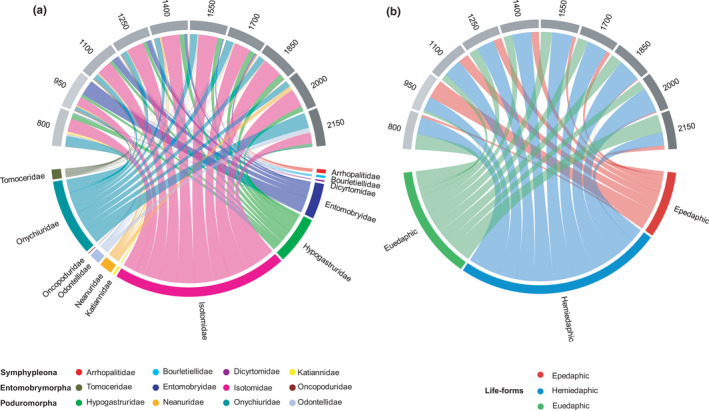
Relative abundance (% of total) of families (a) and life‐forms (b) of Collembola (lower half circle) at 10 altitudes along an altitudinal gradient spanning from 800 to 2150 m at Changbai Mountain (upper half circle). The width of the links represents the relative abundance of families and life‐forms at the respective altitude

Among the 12 families present, Isotomidae dominated (53.4% of total; mean across altitudes), followed by Onychiuridae (16.4%), Hypogastruridae (11.3%), Entomobryidae (7.6%), Neanuridae (4.5%), Tomoceridae (3.0%), and Odontellidae (1.5%) (Figure 2a). Isotomidae predominated at most altitudes (except 950 and 2150 m), with the highest relative abundance at 1850 m (59.3%) and the lowest at 950 m (26.7%) (Figure 3a and Figure S4). The relative abundance of Onychiuridae was highest at 2150 m (44.8%) and the lowest at 950 m (7.7%). The relative abundance of Hypogastruridae averaged 12.7%. The relative abundance of Entomobryidae was high at altitudes between 950 and 1400 m averaging 20.6%, but low at 800 m (1.7%) and between 1550 and 2150 m averaging 1.8%.

Among the three life‐forms, hemiedaphic Collembola dominated (53.5% of total), followed by euedaphic Collembola (29.3%) and epedaphic Collembola (17.2%) (Figure 3b and Figure S5). Hemiedaphic Collembola dominated at each of the altitudes except 950 and 2150 m, and the relative abundance did not differ significantly with altitude (*F* = 1.57, *p* = .157), but was highest at 2000 m (45.0%) and lowest at 2150 m (14.7%). By contrast, epedaphic Collembola varied more strongly with altitude (*F* = 3.13, *p* = .006), and the relative abundance decreased from 950 (64.4%) to 2000 m (30.5%). The relative abundance of euedaphic Collembola was highest at 2150 m (52.2%) and lowest at 950 m (7.7%) but similar between 1100 and 2000 m.

**FIGURE 4:**
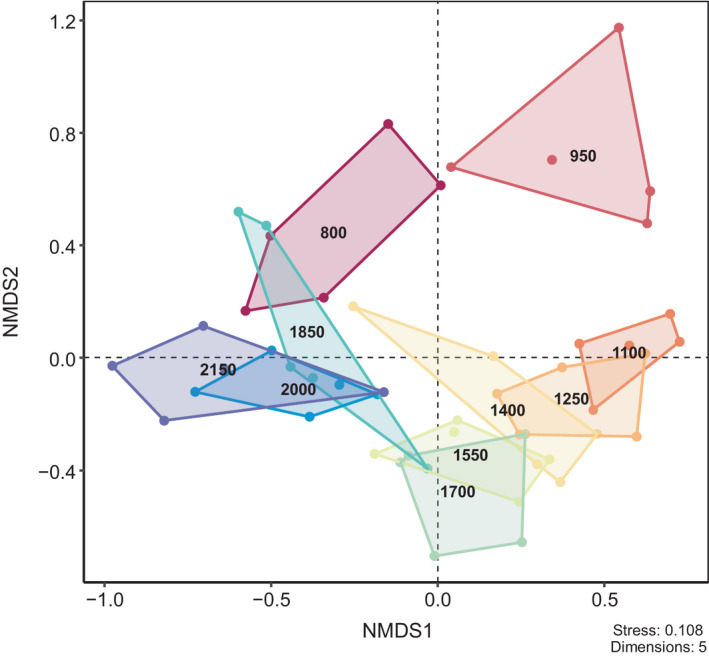
Non‐metric multidimensional scaling (NMDS) ordination based on the Bray–Curtis dissimilarity index of Collembola community composition at ten altitudes along an altitudinal gradient spanning from 800 to 2150 m at Changbai Mountain

Non‐metric multidimensional scaling separated the Collembola communities across altitudes along the first axes with the exception of the site at 800 m, which was positioned similar to the sites between 1850 and 2150 m (Figure 4). The second axis in particular separated the sites at 800 and 950 m from the other sites at higher altitude. Overall, NMDS reflected that Collembola community composition at high altitude sites between 1850 and 2150 m and sites at intermediate altitude between 1100 m and 1700 m was similar and distinct from that at low altitude (800 and 950 m).

### 3.3 Relationship with local habitat‐related factors and climatic variables

**FIGURE 5:**
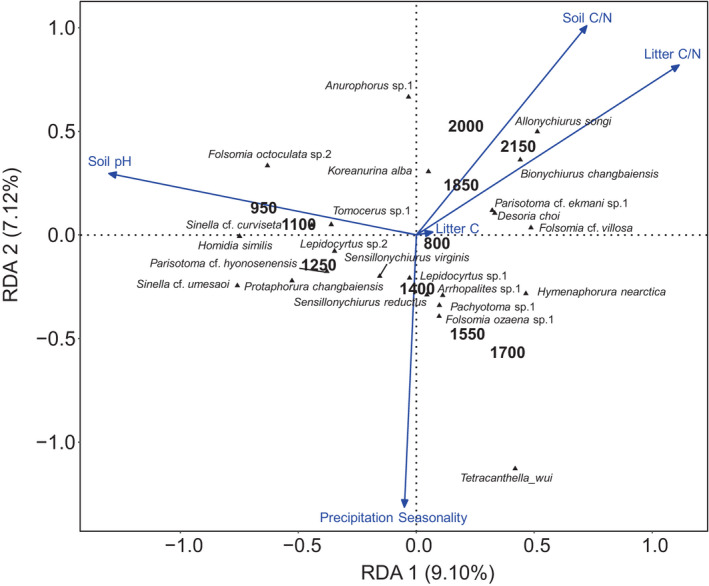
Redundancy analysis of Collembola species along an altitudinal gradient spanning from 800 to 2150 m at Changbai Mountain as related to local habitat‐related factors (soil C/N ratio, litter C/N ratio, and litter C concentration) and climatic variables (annual precipitation, precipitation seasonality, and mean diurnal temperature range) (blue arrows). The length of arrows represents the percentage of variation explained by the respective variable. The 23 species most closely correlating with the first two axes are displayed

Five of the 14 local habitat‐related and climatic variables studied correlated significantly with species composition (RDA, forward selection, overall Monte Carlo test, *p* = .001), together explaining 16.2% of the variation (Figure 5). Soil C/N and litter C/N correlated positively with Collembola communities at high altitudes (1850, 2000, and 2150 m) and were associated with high densities of certain euedaphic (*Allonychiurus songi* and *Bionychiurus changbaiensis*), hemiedaphic (*Anurophorus* sp.1 and *Parisotoma* cf. *ekmani* sp.1), and epedaphic (*Desoria choi* and *Koreanurina alba*) species. Collembola communities at altitudes between 1250 and 1700 m were correlated with precipitation seasonality and high densities of certain euedaphic (*Hymenaphorura nearctica*, *Sensillonychiurus reductus*, *Sensillonychiurus virginis*, and *Protaphorura changbaiensis*), hemiedaphic (*Pachyotoma* sp.1, *Parisotoma* cf. *hyonosenensis*, and *Folsomia ozeana* sp.1), and epedaphic (*Tetracanthella wui*, *Sinella* cf. *umesaoi*, *Arrhopalites* sp.1, and *Lepidocyrtus* sp.1) species. Collembola communities at 950 and 1100 m correlated with soil pH and high densities of epedaphic (*Homidia similis*, *Sinella* cf. *curviseta*, and *Tomocerus* sp.1) and hemiedaphic (*Folsomia octoculata* sp.2) species.

Variation partitioning further indicated that local habitat‐related factors including soil pH, soil C/N, litter C/N, and litter C explained 12.5% of the total variation in Collembola community composition (*p* = .001), while climatic factors (precipitation seasonality) only explained 2.6% of the variation (*p* = .001); climatic and local habitat‐related factors jointly explained 1.6% of the variation.

## 4 DISCUSSION

### 4.1 Changes in density and species richness of Collembola with altitude

Supporting hypothesis 1, species richness of Collembola along the altitudinal gradient at Changbai Mountain followed a hump‐shaped pattern, which has been found before not only for Collembola but also for other soil mesofauna taxa (Jiang et al., 2015). Loranger et al. (2001) also found a similar pattern of Collembola species richness across an altitudinal transect from 950 to 2150 m in the French Alps (temperate forests). In our study, the mid‐peak was at 1100 m, the transition zone between mixed coniferous and broad‐leaved forests, an area with high tree species diversity (Sang & Bai, 2009). This suggests that Collembola species richness is associated with plant species composition as proposed earlier (Maunsell et al., 2013; Sabais et al., 2011). Vegetation characteristics determine soil properties and food supply for microarthropods, thereby likely affecting Collembola richness (Wardle et al., 2004). Indeed, microbial biomass serving as important food resource for Collembola was shown to be higher in the transition zone between mixed coniferous and broad‐leaved forests than in other forest types at Changbai Mountain (Liu et al., 2019).

Contrasting hypothesis 1, the density of Collembola did not change consistently with altitude, with the density in litter exceeding that in soil at most altitudinal zones. This is consistent with previous observations that typically more Collembola (and other soil invertebrates) colonize the litter than the soil layer (Illig et al., 2010; Ma et al., 2020; Mayvan et al., 2015). The decline in density and diversity of Collembola with soil depth may be due to lower availability and quality of food resources in soil than in litter (Illig et al., 2010). Interestingly, the density showed two peaks, one at 1100 m, which was consistent with species richness, but there was an additional peak at 2000 m (not present in species richness). This was because some species of the genera *Folsomia* and *Ceratophysella* reached high densities at 2000 m suggesting them to be well adapted to harsh environmental conditions at high altitudes.

### 4.2 Changes in Collembola community composition with altitude

We hypothesized that community composition of Collembola changes gradually with altitude (hypothesis 2), and our results confirmed this hypothesis at different taxonomic and life‐form levels. At family level, Isotomidae, Onychiuridae, and Hypogastruridae dominated across our study sites and the density of Isotomidae and Hypogastruridae gradually changed with altitude. Similar results have been reported from an altitudinal transect in Russia by Stebaeva’s (2003). In our study, Isotomidae predominated at most altitudes, mainly comprising the genera *Folsomia*, *Heteroisotoma*, and *Desoria*. These eurytopic taxa inhabit soil and litter, but typically are most numerous at moist and cold sites at higher latitude (Potapov 2001). The abundance of Onychiuridae, the second most abundant Collembola group, did not change significantly with altitude, and this is consistent with the findings of Sun et al. (2020) based on sampling a narrower altitudinal gradient from 800 to 1700 m at Changbai Mountain. Onychiuridae comprise uniformly euedaphic species living in soil and therefore were being less exposed to environmental harshness than epedaphic and hemiedaphic species, and this likely contributes to their uniform high abundance across altitudes. The high density of Hypogastruridae was mainly due to the genus *Ceratophysella*, and species of this genus were also found to be among the most abundant Collembola across temperate forests along an altitudinal transect in Mexico (2750–3700 m; García‐Gómez et al., 2009), suggesting that *Ceratophysella* species are well adapted to a wide range of environmental conditions.

Similar to Collembola families, the relative abundance of Collembola life‐forms changed markedly with altitude, with more euedaphic species at higher altitude. Euedaphic species inhabit the mineral soil buffered against climatic harshness. In fact, mineral soil layers at high altitude stay consistently more wet and temperature fluctuates less than at low altitude at Changbai Mountain (Qian et al., 2014), and this likely favors euedaphic species (Berg & Bengtsson, 2007; Heiniger et al., 2015; Parisi et al., 2005). In contrast to euedaphic species, the abundance of hemiedaphic species, which dominated at most altitudes, did not differ significantly between altitudes. Hemiedaphic species colonize both litter and mineral soil, on the one side allowing them to exploit rich litter resources, but on the other to escape drought and frost by retreating into deeper soil layers, which may contribute to their uniform dominance across the studied altitudinal gradient.

Collembola species composition was similar at intermediate (1100–1700 m) and at high (1850–2150 m) altitudes, but differed widely across altitudes. Vegetation at 1100–1700 m was similar comprising subalpine mixed coniferous forest, whereas high altitude sites (1850–2150 m) comprise birch forests or alpine tundra. Similar species composition at both of these vegetation types suggests that they provide similar habitat conditions for Collembola. However, we also found that Collembola communities along the studied altitudinal gradient changed gradually along the first and second NMDS axes, with those at high altitudes (1850, 2000, and 2150 m) being similar to those at the lowest altitude (800 m). In fact, species such as *Allonychiurus songi*, *Ceratophysella* sp.1, *Folsomia* cf. *villosa*, *Folsomia octoculata* sp.2, *Heteraphorura seolagensis*, and *Hymenaphorura nearctica* were abundant at both the high and low altitude sites and thereby contributed to the observed pattern. Moreover, the Onychiuridae and *Folsomia* species dominant at both the high and low altitude sites also reach high abundance in the high Tatra Mountains (Ová et al., 2014), the western Putorana Plateau (Babenko, 2002), and the French northern Alps (Loranger et al., 2001), suggesting that they are well adapted to a wide range of environmental factors in cold and temperate ecosystems. Compared with sites between 1100 and 1700 m, species composition at 800 and 950 m differed along the second axis, which was mainly due to the genera *Semicerura* and *Tetracanthella* reaching high densities at sites between 1100 and 1700 m (Potapov et al., 2020; Xie et al., 2019).

### 4.3 Relationship with local habitat‐related factors and climatic variables

Collembola are known to sensitively respond to soil characteristics (Loranger et al., 2001; Salamon et al., 2008), and we found soil pH, soil C/N ratio, litter C/N ratio, and litter carbon content to significantly correlate with Collembola species composition supporting hypothesis 3. Both euedaphic species (*Allonychiurus songi* and *Bionychiurus changbaiensis*) and epedaphic species (*Desoria choi* and *Koreanurina alba*) were abundant at high altitude sites characterized by high soil and litter C/N ratio, reflecting low quality of organic matter. The results are in line with those of the study of Hasegawa and Takeda (1995), showing that the amount and composition of soil organic matter are important factors driving Collembola community composition. High C/N ratio and low litter quality reflect slow decomposition processes of litter and the formation of thick organic layers known to be important for microarthropods including Collembola (Marian et al., 2018). Moreover, thick organic layers typically are associated with low soil pH (Chagnon et al., 2001; Loranger et al., 2001; Salamon et al., 2008) and this was also the case at our study sites. Soil pH has been shown to drive bacterial community composition at Changbai Mountain (Shen et al., 2013), thereby likely also affecting Collembola feeding microorganisms (Petersen, 1994; Visser, 1985).

Besides local habitat‐related characteristics, climatic factors, such as precipitation seasonality, significantly correlated with Collembola community composition, in part supporting hypothesis 3. This suggests that precipitation directly and/or indirectly structure Collembola community composition, as suggested earlier (Irmler, 2006). At intermediate altitudes (between 1250 and 1700 m), euedaphic (*Hymenaphorura nearctica*, *Sensillonychiurus reductus*, and *Protaphorura changbaiensis*), hemiedaphic (*Pachyotoma* sp.1, *Parisotoma* cf. *hyonosenensis*, and *Folsomia ozeana* sp.1), and epedaphic (*Tetracanthella wui*, *Sinella* cf. *umesaoi*, *Arrhopalites* sp.1, and *Lepidocyrtus* sp.1) species correlated with precipitation seasonality. The precipitation seasonality is also significantly correlated with temperature (Figure S6). This supports earlier suggestions that epedaphic and hemiedaphic species are more vulnerable to increasing temperature and precipitation than euedaphic species, presumably due to epedaphic species predominantly living in litter, thereby being less buffered against temperature and precipitation fluctuations than euedaphic species living in soil. For example, fast development of eggs of epedaphic species may contribute to their high temperature sensitivity (van Straalen, 1994). Notably, *Tetracanthella wui*, a species only reported from Changbai Mountain (Xie et al., 2019), correlated with high precipitation seasonality, suggesting that it can cope with the large precipitation fluctuations occurring at 1400 and 1700 m at Changbai Mountain.

Climatic variables explained less variation in Collembola assemblages than local habitat‐related factors, contrasting our hypothesis 4. This is unlike the study of Xu et al. (2017) stressing that climatic variables are the most critical factor driving diversity patterns of litter‐dwelling invertebrates including Collembola, Acariformes, Parasitiformes, and Diptera across altitude. Local habitat‐related factors likely reflect the availability of food resources, which likely affect the density of soil animals (Ponge, 2000). In addition, Stange and Ayres (2010) found that climatic factors may change insect distribution not only directly through physiological limits due to temperature, precipitation, and humidity, but also indirectly by determining vegetation type and plant abundance, as well as by influencing species interactions. However, hampering the identification of the role of individual factors for Collembola community composition, climatic factors correlated closely with altitude (Figure S6). This corresponds to the suggestion of Li et al. (2020) that altitude is a proxy for a number of environmental factors that directly influence soil fauna distribution. To improve the mechanistic understanding of the role of climatic and local habitat‐related factors in driving belowground communities across altitudes, future research needs to consider additional information on other abiotic environmental and biotic factors (e.g., soil moisture, vegetation characteristics, litter quality and quantity, fungal biomass, and predators), and integrate local factors into a broad geographic sampling design. Ultimately, experimental manipulations will be necessary to identify the role of individual factors in structuring belowground communities along altitudinal gradients.

## CONFLICT OF INTEREST

The authors declare no conflict of interest.

## AUTHOR CONTRIBUTION

**Zhijing Xie:** Data curation (equal); Formal analysis (equal); Investigation (equal); Software (equal); Validation (equal); Visualization (equal); Writing – original draft (equal). **Xin Sun:** Conceptualization (equal); Supervision (equal); Writing – review & editing (equal). **Johannes Lux:** Writing – review & editing (equal). **Ting‐Wen Chen:** Writing – review & editing (equal). **Mikhail Potapov:** Data curation (equal); Investigation (equal). **Donghui Wu:** Conceptualization (equal); Supervision (equal); Writing – review & editing (equal). **Stefan Scheu:** Conceptualization (equal); Supervision (equal); Writing – review & editing (equal).

## Supporting information

Fig S1‐S6

Table S1‐S2

## Data Availability

The data sets analyzed during the current study are available in the Zenodo repository at https://doi.org/10.5281/zenodo.5825701.

## References

[ece38559-bib-0001] Antonelli, A. , Kissling, W. D. , Flantua, S. G. A. , Bermúdez, M. A. , Mulch, A. , Muellner‐Riehl, A. N. , Kreft, H. , Linder, H. P. , Badgley, C. , Fjeldså, J. , Fritz, S. A. , Rahbek, C. , Herman, F. , Hooghiemstra, H. , & Hoorn, C. (2018). Geological and climatic influences on mountain biodiversity. Nature Geoscience, 11, 718–725. 10.1038/s41561-018-0236-z

[ece38559-bib-0002] Babenko, A. (2002). Springtails of western putorana plateau (Middle Siberia): Fauna and altitude differentiation of assemblages. Entomological Review, 82, 901–919.

[ece38559-bib-0003] Bai, F. , Sang, W. , & Axmacher, J. C. (2011). Forest vegetation responses to climate and environmental change: A case study from Changbai Mountain, NE China. Forest Ecology and Management, 262, 2052–2060. 10.1016/j.foreco.2011.08.046

[ece38559-bib-0005] Berg, M. P. , & Bengtsson, J. (2007). Temporal and spatial variability in soil food web structure. Oikos, 116, 1789–1804. 10.1111/j.2007.0030-1299.15748.x

[ece38559-bib-0006] Bokhorst, S. , (Ciska) Veen, G. F. , Sundqvist, M. , De Long, J. R. , Kardol, P. , & Wardle, D. A. (2018). Contrasting responses of springtails and mites to elevation and vegetation type in the sub‐Arctic. Pedobiologia, 67, 57–64. 10.1016/j.pedobi.2018.02.004

[ece38559-bib-0007] Borcard, D. , Legendre, P. , & Drapeau, P. (1992). Partialling out the spatial component of ecological variation. Ecology, 73, 1045–1055. 10.2307/1940179

[ece38559-bib-0008] Brehm, G. , Homeier, J. , & Fiedler, K. (2003). Beta diversity of geometrid moths (Lepidoptera: Geometridae) in an Andean montane rainforest. Diversity and Distributions, 9, 351–366. 10.1046/j.1472-4642.2003.00023.x

[ece38559-bib-0009] Brehm, G. , Strutzenberger, P. , & Fiedler, K. (2013). Phylogenetic diversity of geometrid moths decreases with elevation in the tropical Andes. Ecography, 36, 1247–1253. 10.1111/j.1600-0587.2013.00030.x

[ece38559-bib-0053] Brian, G. P. , & Peter, C. (2020). Performance analytics: Econometric tools for performance and risk analysis. R package version 2.0.4. https://CRAN.R‐project.org/package=PerformanceAnalytics

[ece38559-bib-0010] Chagnon, M. , Paré, D. , Hébert, C. , & Camiré, C. (2001). Effects of experimental liming on collembolan communities and soil microbial biomass in a southern Quebec sugar maple (*Acer saccharum* marsh.) stand. Applied Soil Ecology, 17, 81–90. 10.1016/S0929-1393(00)00134-7

[ece38559-bib-0011] Christiansen, K. , & Bellinger, P. (1998). The collembola of North America, North of the Rio Grande. A taxonomic analysis (2nd edn, pp. 1520). Grinnell College.

[ece38559-bib-0012] Deharveng, L. (2004). Recent advances in Collembola systematics. Pedobiologia, 48, 415–433. 10.1016/j.pedobi.2004.08.001

[ece38559-bib-0013] Dormann, C. F. , Elith, J. , Bacher, S. , Buchmann, C. , Carl, G. , Carré, G. , Marquéz, J. R. G. , Gruber, B. , Lafourcade, B. , Leitão, P. J. , Münkemüller, T. , Mcclean, C. , Osborne, P. E. , Reineking, B. , Schröder, B. , Skidmore, A. K. , Zurell, D. , & Lautenbach, S. (2013). Collinearity: A review of methods to deal with it and a simulation study evaluating their performance. Ecography, 36, 27–46. 10.1111/j.1600-0587.2012.07348.x

[ece38559-bib-0014] Eisenhauer, N. , Sabais, A. C. W. , & Scheu, S. (2011). Collembola species composition and diversity effects on ecosystem functioning vary with plant functional group identity. Soil Biology and Biochemistry, 43, 1697–1704. 10.1016/j.soilbio.2011.04.015

[ece38559-bib-0015] Ferguson, S. H. , & Joly, D. O. (2002). Dynamics of springtail and mite populations: The role of density dependence, predation, and weather. Ecological Entomology, 27, 565–573. 10.1046/j.1365-2311.2002.00441.x

[ece38559-bib-0016] Fick, S. E. , & Hijmans, R. J. (2017). WorldClim 2: new 1‐km spatial resolution climate surfaces for global land areas. International Journal of Climatology, 37, 4302–4315. 10.1002/joc.5086

[ece38559-bib-0017] García‐Gómez, A. , Castaño‐Meneses, G. , & Palacios‐Vargas, J. G. (2009). Diversity of springtails (Hexapoda) according to a altitudinal gradient. Pesquisa Agropecuária Brasileira, 44, 911–916. 10.1590/S0100-204X2009000800016

[ece38559-bib-0018] Gisin, H. (1943). Ökologie und Lebensgemeinschaften der Collembolen im schweizerischen Exkursionsgebiet Basels. Retrieved from http://scholar.google.com/scholar?cluster=1494446119907250369&hl=en&oi=scholarr

[ece38559-bib-0019] Grytnes, J. A. , & McCain, C. M. (2007). Elevational trends in biodiversity. Encyclopedia of Biodiversity, 1–8, 10.1016/b978-012226865-6/00503-1

[ece38559-bib-0020] Guo, Q. , Kelt, D. A. , Sun, Z. , Liu, H. , Hu, L. , Ren, H. , & Wen, J. (2013). Global variation in elevational diversity patterns. Scientific Reports, 3, 3007. 10.1038/srep03007 24157658 PMC6505670

[ece38559-bib-0021] Hasegawa, M. , & Takeda, H. (1995). Changes in feeding attributes of four collembolan populations during the decomposition process of pine needles. Pedobiologia, 39, 155–169.

[ece38559-bib-0022] He, H. S. , Hao, Z. , Mladenoff, D. J. , Shao, G. , Hu, Y. , & Chang, Y. (2005). Simulating forest ecosystem response to climate warming incorporating spatial effects in north‐eastern China. Journal of Biogeography, 32, 2043–2056. 10.1111/j.1365-2699.2005.01353.x

[ece38559-bib-0023] Heiniger, C. , Barot, S. , Ponge, J. F. , Salmon, S. , Meriguet, J. , Carmignac, D. , Suillerot, M. , & Dubs, F. (2015). Collembolan preferences for soil and microclimate in forest and pasture communities. Soil Biology and Biochemistry, 86, 181–192. 10.1016/j.soilbio.2015.04.003

[ece38559-bib-0025] Hodkinson, I. D. (2005). Terrestrial insects along elevation gradients: species and community responses to altitude. Biological Reviews of the Cambridge Philosophical Society, 80(3), 489–513. 10.1017/S1464793105006767 16094810

[ece38559-bib-0026] Hoiss, B. , Krauss, J. , Potts, S. G. , Roberts, S. , & Steffan‐Dewenter, I. (2012). Altitude acts as an environmental filter on phylogenetic composition, traits and diversity in bee communities. Proceedings of the Royal Society B: Biological Sciences, 279, 4447–4456. 10.1098/rspb.2012.1581 PMC3479805 22933374

[ece38559-bib-0027] Hopkin, S. P. (1997). Biology of the springtails (Insecta, Collembola). Oxford University Press.

[ece38559-bib-0028] Hsieh, T. C. , Ma, K. H. , & Chao, A. (2016). iNEXT: an R package for rarefaction and extrapolation of species diversity (Hill numbers). Methods in Ecology and Evolution, 7(12), 1451–1456. 10.1111/2041-210X.12613/FORMAT/PDF

[ece38559-bib-0029] Illig, J. , Norton, R. A. , Scheu, S. , & Maraun, M. (2010). Density and community structure of soil‐ and bark‐dwelling microarthropods along an altitudinal gradient in a tropical montane rainforest. Experimental and Applied Acarology, 52, 49–62. 10.1007/s10493-010-9348-x 20229099 PMC2914295

[ece38559-bib-0030] Irmler, U. (2006). Climatic and litter fall effects on collembolan and oribatid mite species and communities in a beech wood based on a 7 years investigation. European Journal of Soil Biology, 42, 51–62. 10.1016/j.ejsobi.2005.09.016

[ece38559-bib-0031] Jiang, Y. , Yin, X. , & Wang, F. (2015). Composition and spatial distribution of soil mesofauna along an elevation gradient on the north slope of the Changbai Mountains, China. Pedosphere, 25, 811–824. 10.1016/S1002-0160(15)30062-X

[ece38559-bib-0032] Johnson, D. , Krsek, M. , Wellington, E. M. H. , Stott, A. W. , Cole, L. , Bardgett, R. D. , Read, D. J. , & Leake, J. R. (2005). Soil invertebrates disrupt carbon flow through fungal networks. Science, 309, 1047. 10.1126/science.1114769 16099977

[ece38559-bib-0033] Kessler, M. (2001). Patterns of diversity and range size of selected plant groups along an elevational transect in the Bolivian Andes. Biodiversity and Conservation, 10, 1897–1921. 10.1023/A:1013130902993

[ece38559-bib-0034] Krashevska, V. , Bonkowski, M. , Maraun, M. , & Scheu, S. (2007). Testate amoebae (protista) of an elevational gradient in the tropical mountain rain forest of Ecuador. Pedobiologia, 51, 319–331. 10.1016/j.pedobi.2007.05.005

[ece38559-bib-0035] Li, J. , Li, Q. , Wu, Y. , Ye, L. , Liu, H. , Wei, J. , & Huang, X. (2021). Mountains act as museums and cradles for hemipteran insects in China: Evidence from patterns of richness and phylogenetic structure. Global Ecology and Biogeography, 30, 1070–1085. 10.1111/geb.13276

[ece38559-bib-0036] Li, X. , Chen, X. , Zhu, H. , Ren, Z. , Jiao, J. , Hu, F. , & Liu, M. (2020). Effects of historical legacies on soil nematode communities are mediated by contemporary environmental conditions. Ecology and Evolution, 10(13), 6732–6740. 10.1002/ece3.6406 32724546 PMC7381565

[ece38559-bib-0037] Liu, M. , Sui, X. , Hu, Y. , & Feng, F. (2019). Microbial community structure and the relationship with soil carbon and nitrogen in an original Korean pine forest of Changbai Mountain, China. BMC Microbiology, 19, 218. 10.1186/s12866-019-1584-6 31519147 PMC6743161

[ece38559-bib-0038] Lomolino, M. V. (2001). Elevation gradients of species‐density: Historical and prospective views. Global Ecology and Biogeography, 10, 3–13. 10.1046/j.1466-822x.2001.00229.x

[ece38559-bib-0039] Loranger, G. , Bandyopadhyaya, I. , Razaka, B. , & Ponge, J. F. (2001). Does soil acidity explain altitudinal sequences in collembolan communities? Soil Biology and Biochemistry, 33, 381–393. 10.1016/S0038-0717(00)00153-X

[ece38559-bib-0040] Ma, C. , Yin, X. , Xu, H. , & Tao, Y. (2020). Responses of soil Collembolans to vegetation restoration in temperate coniferous and broad‐leaved mixed forests. Journal of Forestry Research, 31, 2333–2345. 10.1007/s11676-019-01005-9

[ece38559-bib-0042] Malhi, Y. , Silman, M. , Salinas, N. , Bush, M. , Meir, P. , & Saatchi, S. (2010). Introduction: Elevation gradients in the tropics: Laboratories for ecosystem ecology and global change research. Global Change Biology, 16(12), 3171–3175. 10.1111/j.1365-2486.2010.02323.x

[ece38559-bib-0043] Marian, F. , Sandmann, D. , Krashevska, V. , Maraun, M. , & Scheu, S. (2018). Altitude and decomposition stage rather than litter origin structure soil microarthropod communities in tropical montane rainforests. Soil Biology and Biochemistry, 125, 263–274. 10.1016/j.soilbio.2018.07.017

[ece38559-bib-0044] Maunsell, S. C. , Kitching, R. L. , Greenslade, P. , Nakamura, A. , & Burwell, C. J. (2013). Springtail (Collembola) assemblages along an elevational gradient in Australian subtropical rainforest. Australian Journal of Entomology, 52, 114–124. 10.1111/aen.12012

[ece38559-bib-0045] Mayvan, M. M. , Shayanmehr, M. , & Scheu, S. (2015). Depth distribution and inter‐annual fluctuations in density and diversity of Collembola in an Iranian Hyrcanian forest. Soil Organisms, 87, 239–247.

[ece38559-bib-0046] McCain, C. M. , & Grytnes, J. A. (2010) Elevational gradients in species richness. In: Encyclopedia of life sciences (pp. 1–10). John Wiley & Sons, Ltd. 10.1002/9780470015902.a0022548

[ece38559-bib-0047] Mertens, J. , Coessens, R. , & Blancquaert, J. P. (1983). Reproduction and development of *Hypogastrura viatica* in relation to temperature and submerged condition. Revue D’ecologie Et De Biologie Du Sol, 20, 567–577.

[ece38559-bib-0048] Oksanen, J. , Blanchet, F. G. , Friendly, M. , Kindt, R. , Legendre, P. , McGlinn, D. , Minchin, P. R. , O’Hara, R. B. , Simpson, G. L. , Solymos, P. , Stevens, M. H. H. , Szoecs, E. , & Wagner, H. (2019). vegan: Community Ecology Package. R package version 2.5‐2. Cran R 1, 2. https://cran.r‐project.org/package=vegan

[ece38559-bib-0049] Ová, V. U. Č. , Miklisová, D. , & Ková, Ľ. Č. (2014). Forest disturbance enhanced the activity of epedaphic collembola in windthrown stands of the high tatra mountains. Journal of Mountain Science, 11, 449–463. 10.1007/s11629-013-2736-z

[ece38559-bib-0050] Parisi, V. , Menta, C. , Gardi, C. , Jacomini, C. , & Mozzanica, E. (2005). Microarthropod communities as a tool to assess soil quality and biodiversity: A new approach in Italy. Agriculture, Ecosystems and Environment, 105, 323–333. 10.1016/j.agee.2004.02.002

[ece38559-bib-0051] Pauli, H. , Gottfried, M. , Dullinger, S. , Abdaladze, O. , Akhalkatsi, M. , Alonso, J. L. B. , Coldea, G. , Dick, J. , Erschbamer, B. , Calzado, R. F. , Ghosn, D. , Holten, J. I. , Kanka, R. , Kazakis, G. , Kollár, J. , Larsson, P. , Moiseev, P. , Moiseev, D. , Molau, U. , … Grabherr, G. (2012). Recent plant diversity changes on Europe’s mountain summits. Science, 336(6079), 353–355. 10.1126/science.1219033 22517860

[ece38559-bib-0052] Peng, Y. , Holmstrup, M. , Schmidt, I. K. , De Schrijve, A. , Schelfhout, S. , Heděnec, P. , Zheng, H. , Bachega, L. R. , Yue, K. , & Vesterdal, L. (2022). Litter quality, mycorrhizal association, and soil properties regulate effects of tree species on the soil fauna community. Geoderma, 407, 115570. 10.1016/j.geoderma.2021.115570

[ece38559-bib-0092] Petersen, H. (1994). A review of collembolan ecology in ecosystem context. Acta Zoologica Fennica, 195, 111–118.

[ece38559-bib-0055] Ponge, J. F. (2000). Vertical distribution of Collembola (Hexapoda) and their food resources in organic horizons of beech forests. Biology and Fertility of Soils, 32(6), 508–522. 10.1007/s003740000285

[ece38559-bib-0057] Potapov, M. (2001). Synopses on Palaearctic Collembola vol. 3: Isotomidae. Abhandlungen und Berichte des Naturkundemuseums Görlitz, 73, 1–603.

[ece38559-bib-0058] Potapov, M. , Xie, Z. , Kuprin, A. , & Sun, X. (2020). The genus Semicerura (Collembola; Isotomidae) in Asia. Zootaxa, 4751(1), 105–118. 10.11646/zootaxa.4751.1.5 32230433

[ece38559-bib-0059] Qian, H. , Hao, Z. , & Zhang, J. (2014). Phylogenetic structure and phylogenetic diversity of angiosperm assemblages in forests along an elevational gradient in Changbaishan, China. Journal of Plant Ecology, 7, 154–165. 10.1093/jpe/rtt072

[ece38559-bib-0060] R Core Team . (2021). R: A language and environment for statistical computing. R Core Team.

[ece38559-bib-0061] Rahbek, C. (2005). The role of spatial scale and the perception of large‐scale species‐richness patterns. Ecology Letters, 8, 224–239. 10.1111/j.1461-0248.2004.00701.x

[ece38559-bib-0091] Robert, J. H. (2021). raster: Geographic Data Analysis and Modeling. R package version 3.4‐10. https://CRAN.R‐project.org/package=raster

[ece38559-bib-0062] Russell, L. (2020). Emmeans: Estimated Marginal Means, aka Least‐Squares Means. R Package version 1.4.4. https://CRAN.R‐project.org/package=emmeans

[ece38559-bib-0063] Sabais, A. C. W. , Eisenhauer, N. , König, S. , Renker, C. , Buscot, F. , & Scheu, S. (2012). Soil organisms shape the competition between grassland plant species. Oecologia, 170, 1021–1032. 10.1007/s00442-012-2375-z 22678109

[ece38559-bib-0064] Sabais, A. C. W. , Scheu, S. , & Eisenhauer, N. (2011). Plant species richness drives the density and diversity of Collembola in temperate grassland. Acta Oecologica, 37, 195–202. 10.1016/j.actao.2011.02.002

[ece38559-bib-0065] Salamon, J. A. , Scheu, S. , & Schaefer, M. (2008). The Collembola community of pure and mixed stands of beech (*Fagus sylvatica*) and spruce (*Picea abies*) of different age. Pedobiologia, 51, 385–396. 10.1016/j.pedobi.2007.10.002

[ece38559-bib-0067] Sang, W. , & Bai, F. (2009). Vascular diversity patterns of forest ecosystem before and after a 43‐year interval under changing climate conditions in the Changbaishan Nature Reserve, northeastern China. Forest Ecology: Recent Advances in Plant Ecology, 210, 115–130, 10.1007/978-90-481-2795-5_10

[ece38559-bib-0068] Scheu, S. , Illig, J. , Eissfeller, K. V. , Sandmann, D. , & Maraun, M. (2008). The soil fauna of a tropical mountain rainforest in southern Ecuador: Structure and functioning. In: Tropical mountain forest: Patterns and processes in a biodiversity hotspot, Biodiversity and Ecology Series Vol. 2, (pp. 79–96).

[ece38559-bib-0070] Shen, C. , Liang, W. , Shi, Y. , Lin, X. , Zhang, H. , Wu, X. , Xie, G. , Chain, P. , Grogan, P. , & Chu, H. (2014). Contrasting elevational diversity patterns between eukaryotic soil microbes and plants. Ecology, 95, 3190–3202. 10.1890/14-0310.1

[ece38559-bib-0071] Shen, C. , Xiong, J. , Zhang, H. , Feng, Y. , Lin, X. , Li, X. , Liang, W. , & Chu, H. (2013). Soil pH drives the spatial distribution of bacterial communities along elevation on Changbai Mountain. Soil Biology and Biochemistry, 57, 204–211. 10.1016/j.soilbio.2012.07.013

[ece38559-bib-0072] Snider, R. J. , & Butcher, J. W. (1972). Response of *Onychiurus justi* (Denis) (Collembola: Onychiuridae) to constant temperatures and variable relative humidity. In Proceedings of the First Soil Microcommunities Conference, USAEC, Syracuse, New York, pp. 176–184.

[ece38559-bib-0073] Stange, E. E. , & Ayres, M. P. (2010). Climate Change Impacts: Insects. Encyclopedia of Life Sciences, 1–7, 10.1002/9780470015902.a0022555

[ece38559-bib-0074] Stebaeva, S. (2003). Collembolan communities of the Ubsu‐Nur Basin and adjacent mountains (Russia, Tuva). Pedobiologia, 47, 341–356. 10.1078/0031-4056-00198

[ece38559-bib-0075] Stone, R. (2006). A threatened nature reserve breaks down Asian borders. Science, 313(5792), 1379–1380. 10.1126/science.313.5792.1379 16959985

[ece38559-bib-0076] Sun, X. , Deharveng, L. , Bedos, A. , Chang, L. , Scheu, S. , & Wu, D. (2020). Changes in diversity and body size of Onychiurinae (Collembola: Onychiuridae) along an altitudinal gradient in Changbai Mountain, China. Soil Ecology Letters, 2, 230–239. 10.1007/s42832-020-0040-8

[ece38559-bib-0077] Traunspurger, W. , Reiff, N. , Krashevska, V. , Majdi, N. , & Scheu, S. (2017). Diversity and distribution of soil micro‐invertebrates across an altitudinal gradient in a tropical montane rainforest of Ecuador, with focus on free‐living nematodes. Pedobiologia, 62, 28–35. 10.1016/j.pedobi.2017.04.003

[ece38559-bib-0078] van Straalen, N. M. (1994). Adaptive significance of temperature responses in Collembola. Acta Zoologica Fennica, 195, 135–142.

[ece38559-bib-0093] Visser, S. (1985). Role of the soil invertebrates in determining the composition of soil microbial communities. In A. H. Fitter & D. Atkinson (Eds.), Biological interactions in soil (pp. 297–317). Blackwell Scientific Publishers.

[ece38559-bib-0079] Wang, L. , Wang, W. J. , Wu, Z. , Du, H. , Zong, S. , & Ma, S. (2019). Potential distribution shifts of plant species under climate change in Changbai Mountains, China. Forests, 10, 1–15. 10.3390/f10060498

[ece38559-bib-0080] Wardle, D. A. , Bardgett, R. D. , Klironomos, J. N. , Setälä, H. , Van Der Putten, W. H. , & Wall, D. H. (2004). Ecological linkages between aboveground and belowground biota. Science, 304(5677), 1629–1633. 10.1126/science.1094875 15192218

[ece38559-bib-0081] Widenfalk, L. A. , Bengtsson, J. , Berggren, Å. , Zwiggelaar, K. , Spijkman, E. , Huyer‐Brugman, F. , & Berg, M. P. (2015). Spatially structured environmental filtering of collembolan traits in late successional salt marsh vegetation. Oecologia, 179, 537–549. 10.1007/s00442-015-3345-z 26001605 PMC4568007

[ece38559-bib-0082] Wu, Y. , & Lei, F. (2013). Species richness patterns and mechanism along the elevational gradients. Chinese Journal of Zoology, 48(5), 797–807. 10.13859/j.cjz.2013.05.022

[ece38559-bib-0083] Xie, Z. , Potapov, M. , & Sun, X. (2019). Two new species of the genus *Tetracanthella* (Collembola: Isotomidae) from China. Zootaxa, 4585, 573–580. 10.11646/zootaxa.4585.3.11 31716163

[ece38559-bib-0094] Xie, Z. J. , Chen, T. W. , Potapov, M. , Zhang, F. , Wu, D. H. , Scheu, S. , & Sun, X. (2022). Ecological and evolutionary processes shape below ground springtail communities along an elevational gradient. Journal of Biogeography, 49(1). 10.1111/jbi.14317

[ece38559-bib-0084] Xu, G. , Lin, Y. , Zhang, S. , Zhang, Y. , Li, G. , & Ma, K. (2017). Shifting mechanisms of elevational diversity and biomass patterns in soil invertebrates at treeline. Soil Biology and Biochemistry, 113, 80–88. 10.1016/j.soilbio.2017.05.012

[ece38559-bib-0085] Xu, G. , Zhang, S. , Lin, Y. , & Ma, K. (2015). Context dependency of the density‐body mass relationship in litter invertebrates along an elevational gradient. Soil Biology and Biochemistry, 88, 323–332. 10.1016/j.soilbio.2015.06.010

[ece38559-bib-0086] Xu, W. D. , He, X. Y. , Chen, W. , & Liu, C. F. (2004). Characteristics and succession rules of vegetation types in Changbai Mountain. Chinese Journal of Ecology, 23, 162–174.

[ece38559-bib-0087] Xue, D. , & Tisdell, C. (2001). Valuing ecological functions of biodiversity in Changbaishan Mountain Biosphere Reserve in Northeast China. Biodiversity and Conservation, 10(3), 467–481. 10.1023/A:1016630825913

[ece38559-bib-0088] Yang, X. , & Xu, M. (2003). Biodiversity conservation in Changbai Mountain Biosphere Reserve, northeastern China: Status, problem, and strategy. Biodiversity and Conservation, 12(5), 883–903. 10.1023/A:1022841107685

[ece38559-bib-0089] Zou, Y. , Sang, W. , Bai, F. , & Axmacher, J. C. (2013). Relationships between plant diversity and the abundance and α‐diversity of predatory ground beetles (coleoptera: Carabidae) in a mature Asian temperate forest ecosystem. PLoS One, 8(12), e82792. 10.1371/journal.pone.0082792 24376582 PMC3869730

[ece38559-bib-0090] Zou, Y. , Sang, W. , Zhou, H. , Huang, L. , & Axmacher, J. C. (2014). Altitudinal diversity patterns of ground beetles (Coleoptera: Carabidae) in the forests of Changbai Mountain, Northeast China. Insect Conservation and Diversity, 7(2), 161–171. 10.1111/icad.12039

