# Supplementary figures and images for: Drivers of Collembola assemblages along an altitudinal gradient in northeast China
Source: Ecol Evol. 2022 Feb 12;12(2):e8559. doi: 10.1002/ece3.8559 (PMC8840876; doi:10.1002/ece3.8559)

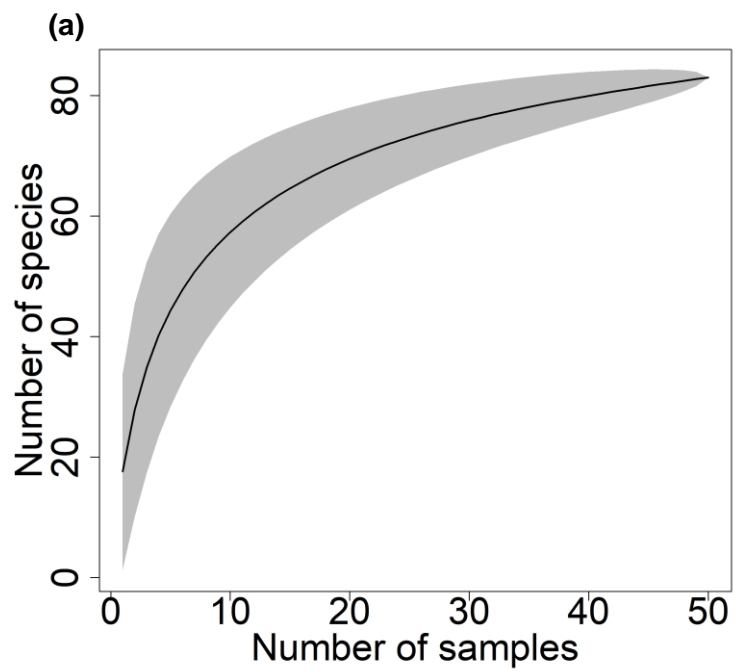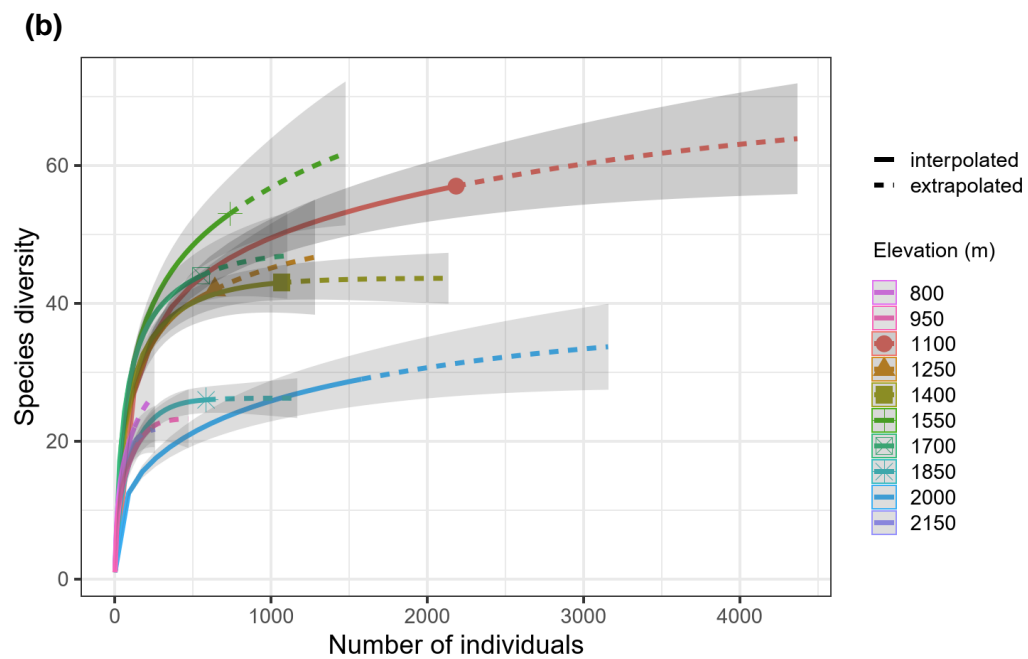

**Figure S1.**

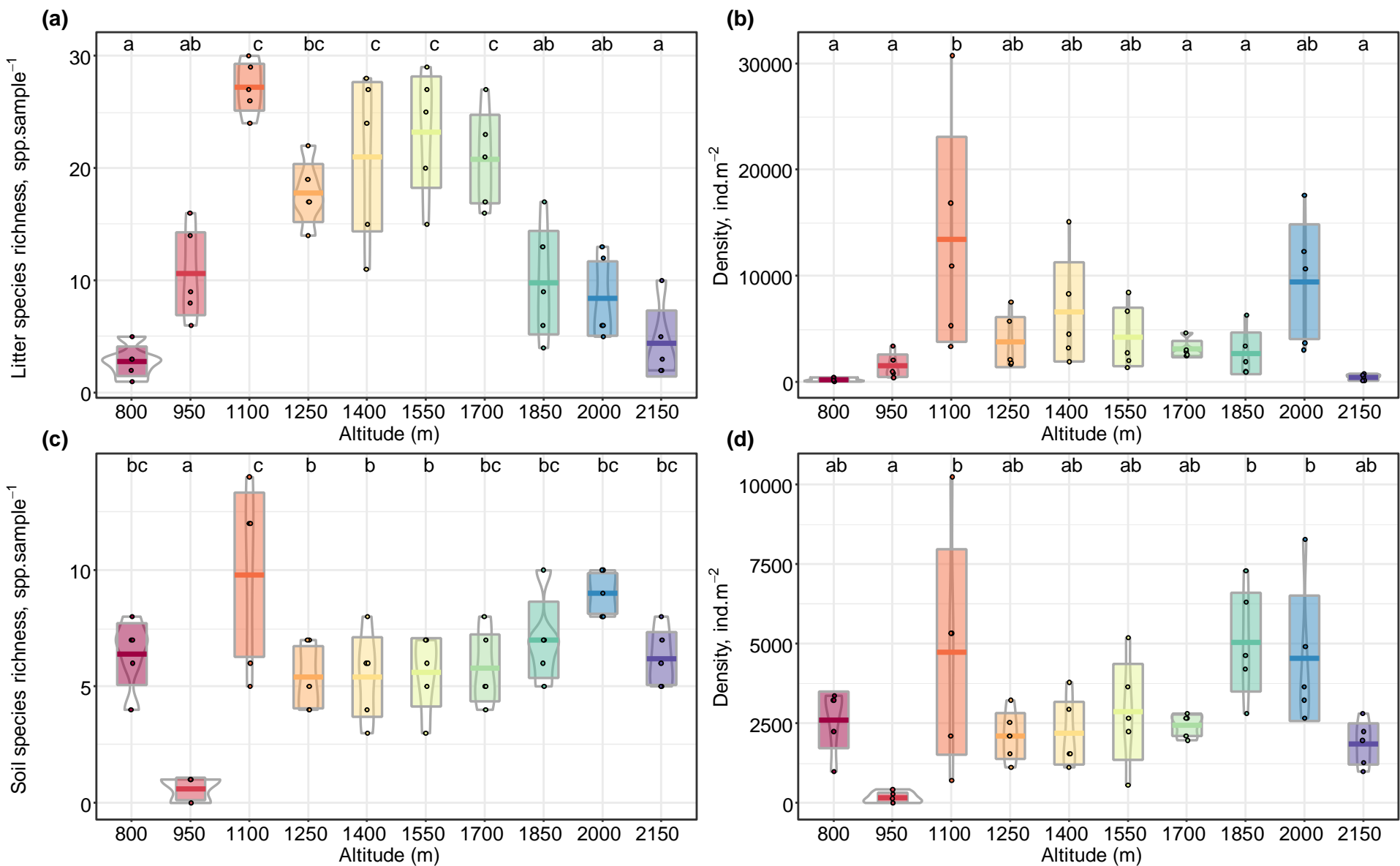

**Figure S2.**

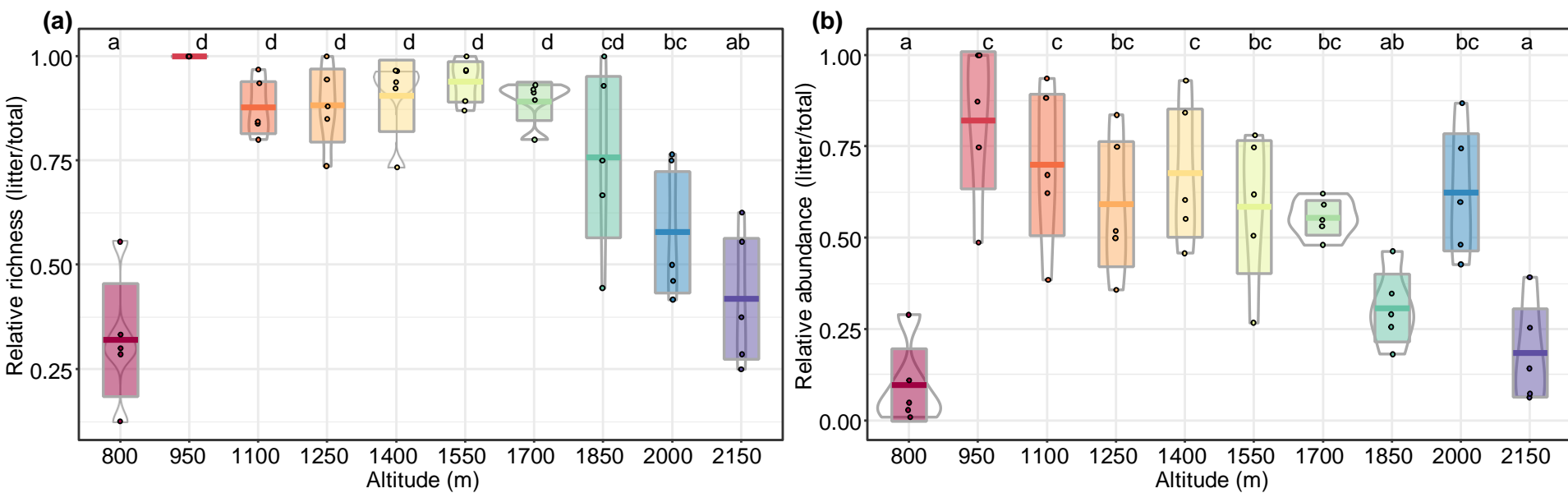

**Figure S3.**

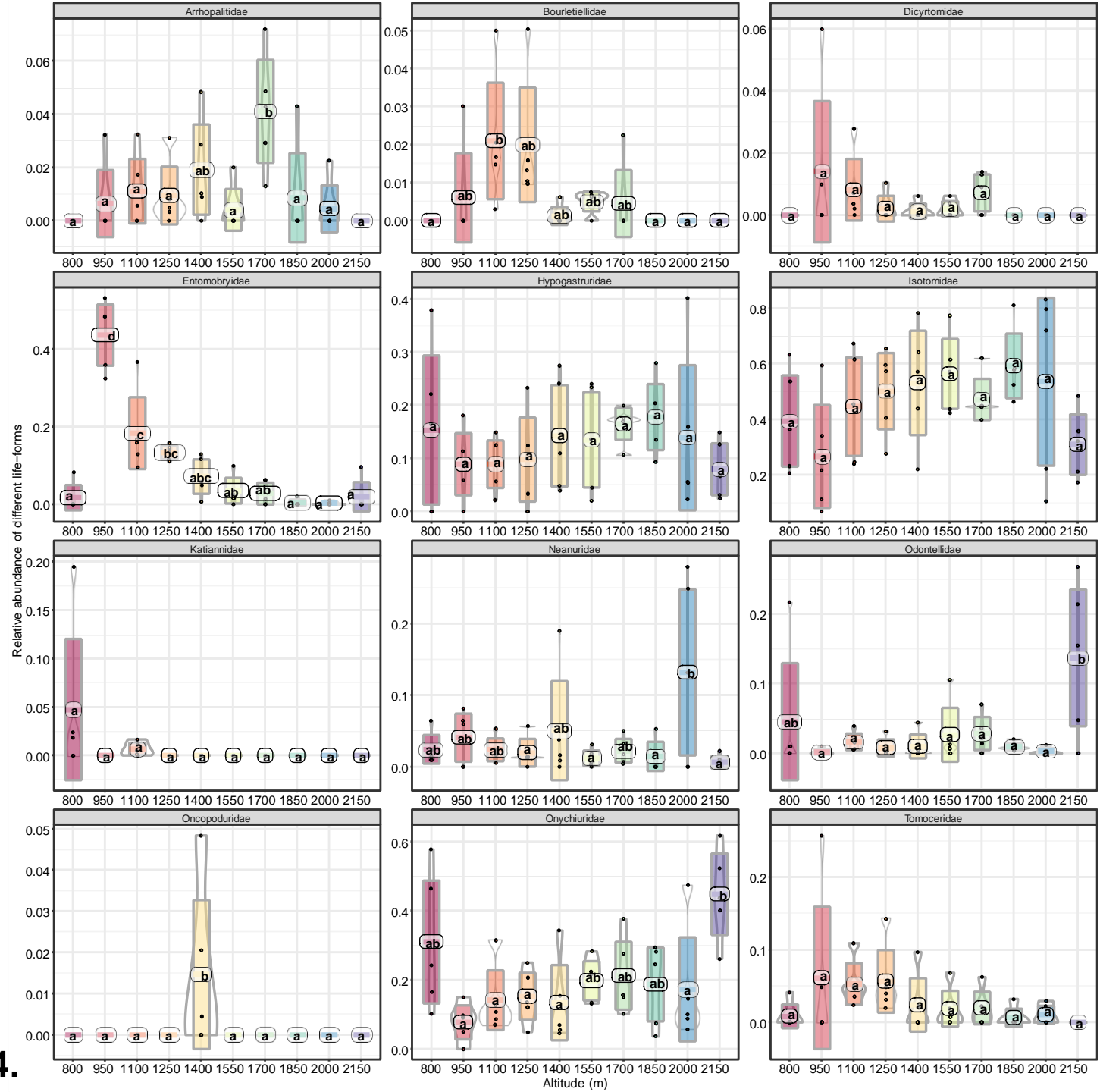

**Figure S4.**

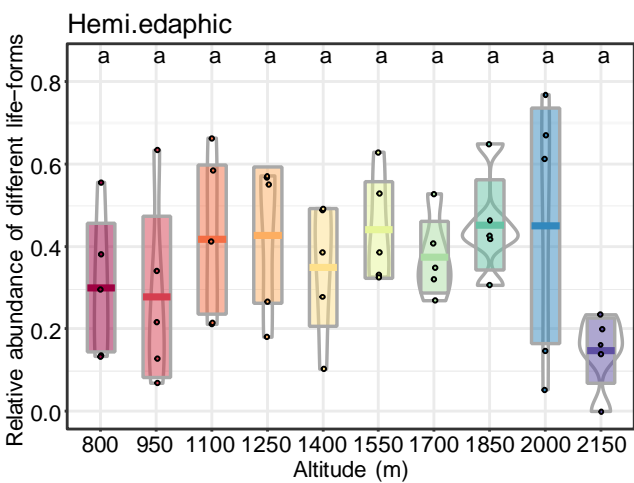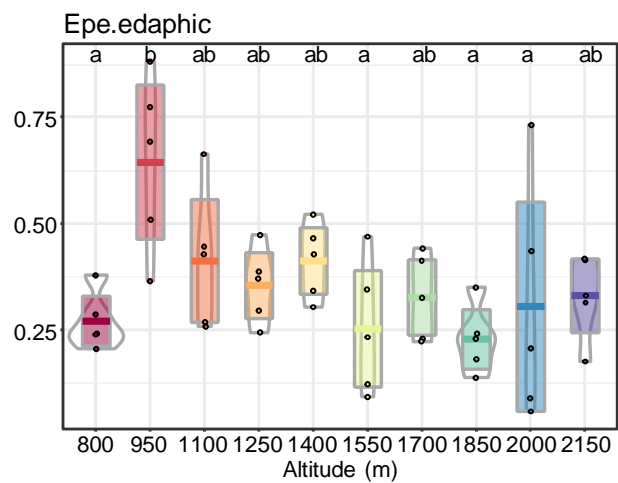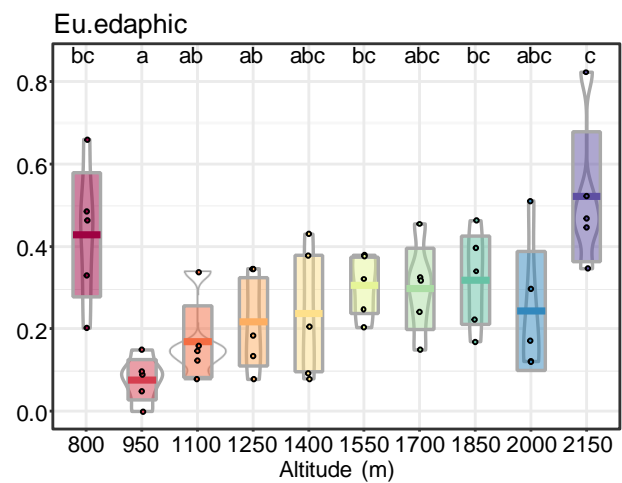

**Figure S5.**

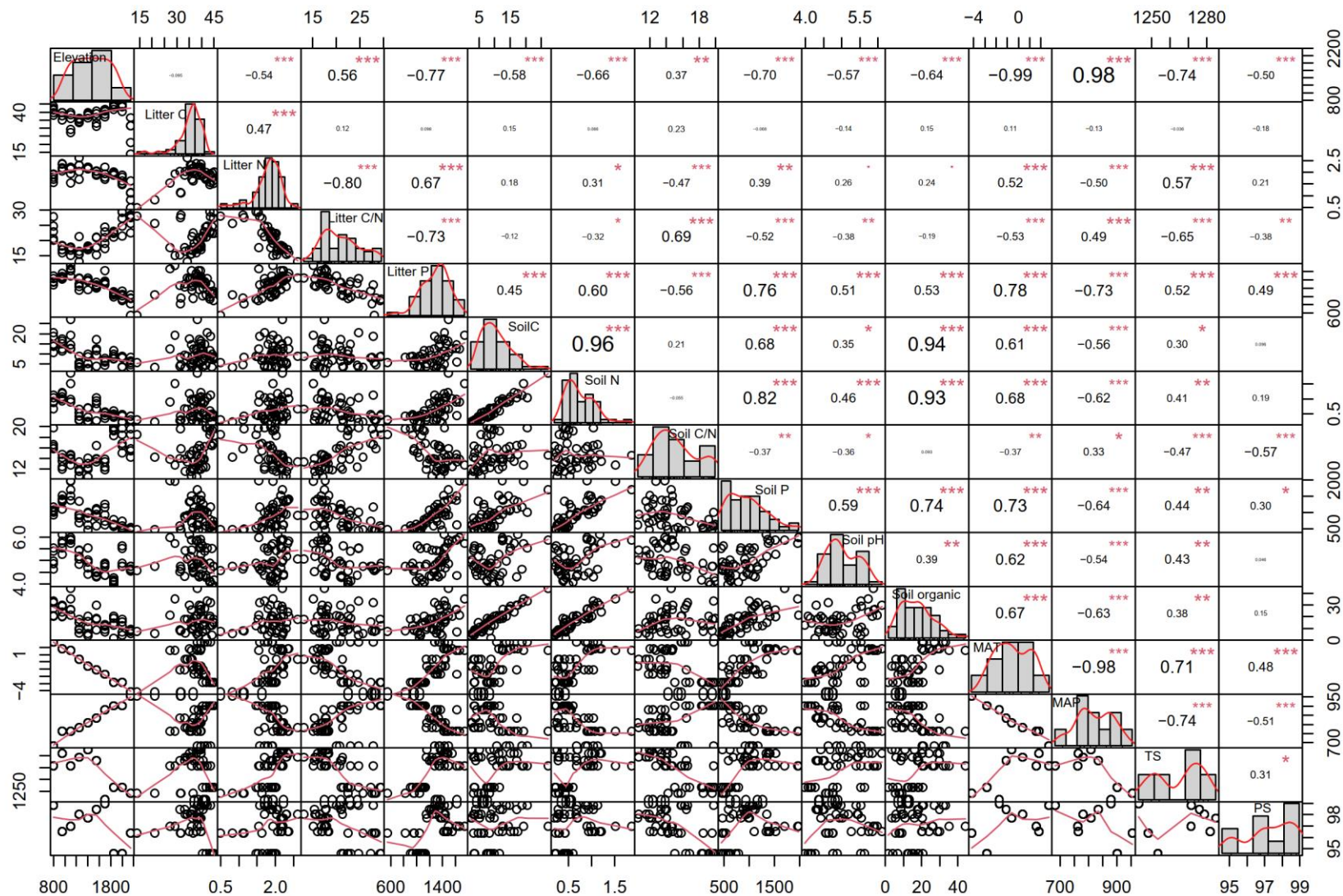

Figure S6.

Supplement: Supplementary file 1 — Fig S1‐S6 [file ECE3-12-e8559-s002.pdf]
