# Supplementary material for: Drivers of Collembola assemblages along an altitudinal gradient in northeast China
Source: Ecol Evol. 2022 Feb 12;12(2):e8559. doi: 10.1002/ece3.8559 (PMC8840876; doi:10.1002/ece3.8559)
Supplement: Supplementary file 2 — Table S1‐S2 [file ECE3-12-e8559-s001.docx]

**Table S1.** The mean values of environmental factors of the ten altitudes.

| **Elevation (m)** | **Litter C (%)** | **Litter N (%)** | **Litter C/N** | **Litter P (mg/kg)** | **Soil C (%)** | **Soil N (%)** | **Soil C/N** | **Soil P (mg/kg)** | **Soil pH** | **Soil Organic (%)** | **MAT** | **MAP** | **TS** | **PS** |
| --- | --- | --- | --- | --- | --- | --- | --- | --- | --- | --- | --- | --- | --- | --- |
| 800 | 41,02 | 1,78 | 23,27 | 1382,19 | 15,72 | 0,93 | 16,85 | 1046,67 | 5,08 | 26,62 | 2.71 | 683,00 | 1271,65 | 98,82 |
| 950 | 38,41 | 2,22 | 17,33 | 1470,01 | 14,53 | 1,08 | 13,37 | 1351,12 | 5,70 | 24,60 | 1.82 | 764,00 | 1281,95 | 96,51 |
| 1100 | 34,09 | 2,01 | 16,99 | 1626,90 | 17,23 | 1,23 | 13,99 | 1706,95 | 5,87 | 30,50 | 1.58 | 762,00 | 1271,47 | 96,97 |
| 1250 | 35,07 | 2,18 | 16,43 | 1313,14 | 5,24 | 0,44 | 11,80 | 944,61 | 5,19 | 11,46 | 0.36 | 782,00 | 1278,80 | 98,02 |
| 1400 | 38,31 | 2,18 | 17,58 | 1350,93 | 5,73 | 0,45 | 12,36 | 781,10 | 4,92 | 11,29 | -0.61 | 809,00 | 1285,04 | 97,70 |
| 1550 | 36,17 | 1,95 | 18,59 | 1260,03 | 10,92 | 0,76 | 14,30 | 923,10 | 4,40 | 18,53 | -1.11 | 835,00 | 1276,05 | 98,47 |
| 1700 | 39,81 | 1,92 | 20,97 | 1384,84 | 6,22 | 0,49 | 12,76 | 941,67 | 4,64 | 10,72 | -1.72 | 880,00 | 1242,88 | 98,97 |
| 1850 | 43,57 | 1,95 | 22,31 | 1065,61 | 9,08 | 0,47 | 19,31 | 640,98 | 4,12 | 13,60 | -2.83 | 902,00 | 1253,40 | 94,58 |
| 2000 | 43,47 | 1,66 | 26,47 | 1010,29 | 6,47 | 0,37 | 17,27 | 558,68 | 5,11 | 11,33 | -2.83 | 902,00 | 1253,40 | 94,58 |
| 2150 | 26,93 | 1,04 | 26,45 | 866,33 | 5,70 | 0,34 | 16,70 | 488,49 | 4,66 | 7,66 | -4.17 | 955,00 | 1248,21 | 96,38 |
|  |  |  |  |  |  |  |  |  |  |  |  |  |  |  |

MAT = Mean Annual Temperature (°C), MAP = Mean Annual Precipitation (mm), TS = Temperature seasonality, PS = Precipitation seasonality.

**Table S2.** Density (with standard deviation, individuals per m^2^) of Collembola morphospecies across the ten altitudes at Changbai Mountain.

| **Life-forms** | **Species Names** | **800 m** | **950 m** | **1100 m** | **1250 m** | **1400 m** | **1550 m** | **1700 m** | **1850 m** | **2000 m** | **2150 m** |
| --- | --- | --- | --- | --- | --- | --- | --- | --- | --- | --- | --- |
| Eu-edaphic | *Allonychiurus songi* | 84±188 | 26±28 | 559±827 | 112±251 | 263±312 | 253±322 | 283±299 | 718±471 | 1086±1435 | 505±404 |
| Hemi-edaphic | *Anurophorus* sp.1 | 0 | 0 | 0 | 40±89 | 0 | 28±63 | 0 | 0 | 4800±5546 | 20±45 |
| Epe-edaphic | *Arrhopalites* sp.1 | 0 | 13±30 | 0 | 27±43 | 113±76 | 20±45 | 240±176 | 56±125 | 28±63 | 0 |
| Epe-edaphic | *Arrhopalites* sp.2 | 0 | 0 | 119±148 | 13±18 | 7±15 | 0 | 0 | 0 | 0 | 0 |
| Eu-edaphic | *Bionychiurus changbaiensis* | 0 | 7±15 | 266±352 | 0 | 96±83 | 268±199 | 33±74 | 119±197 | 343±331 | 308±208 |
| Epe-edaphic | *Bourletiella* sp.1 | 0 | 7±15 | 253±192 | 141±186 | 20±45 | 26±15 | 0 | 0 | 0 | 0 |
| Epe-edaphic | *Bourletiella* sp.2 | 0 | 0 | 20±45 | 7±15 | 0 | 13±18 | 33±74 | 0 | 0 | 0 |
| Hemi-edaphic | *Ceratophysella* cf. *skarzynskii* | 0 | 0 | 28±63 | 0 | 0 | 0 | 0 | 0 | 0 | 0 |
| Hemi-edaphic | *Ceratophysella* sp.1 | 447±425 | 147±96 | 1204±775 | 420±277 | 1199±1003 | 729±503 | 863±196 | 1166±118 | 1902±2205 | 179±135 |
| Hemi-edaphic | *Ceratophysella* sp.2 | 0 | 0 | 0 | 0 | 0 | 26±28 | 40±43 | 0 | 0 | 0 |
| Epe-edaphic | *Desoria* sp.1 | 0 | 0 | 0 | 0 | 0 | 28±63 | 0 | 0 | 0 | 0 |
| Epe-edaphic | *Desoria* sp.2 | 0 | 0 | 0 | 0 | 0 | 0 | 7±15 | 0 | 0 | 0 |
| Epe-edaphic | *Desoria* sp.3 | 0 | 0 | 0 | 0 | 20±45 | 0 | 0 | 0 | 0 | 0 |
| Epe-edaphic | *Desoria* sp.7 | 0 | 0 | 13±30 | 0 | 0 | 7±15 | 0 | 0 | 0 | 28±63 |
| Epe-edaphic | *Desoria* sp.9 | 0 | 0 | 28±63 | 0 | 0 | 20±45 | 13±30 | 0 | 27±59 | 56±77 |
| Epe-edaphic | *Desoria* cf. *nivea* | 0 | 0 | 0 | 0 | 140±313 | 0 | 0 | 0 | 0 | 0 |
| Epe-edaphic | *Desoria choi* | 0 | 0 | 0 | 0 | 460±871 | 80±119 | 33±47 | 88±178 | 117±127 | 116±118 |
| Epe-edaphic | *Desoria tigrina* sp. | 0 | 0 | 0 | 0 | 0 | 0 | 0 | 0 | 7±15 | 0 |
| Hemi-edaphic | *Deutonura* cf. *frigida* | 0 | 0 | 0 | 0 | 0 | 7±15 | 0 | 0 | 0 | 20±30 |
| Hemi-edaphic | *Deutonura* cf. *muscorum* | 73±89 | 13±18 | 280±263 | 27±59 | 20±30 | 7±15 | 41±58 | 0 | 0 | 0 |
| Hemi-edaphic | *Deutonura* sp.1 | 0 | 13±30 | 0 | 0 | 0 | 0 | 7±15 | 27±59 | 0 | 0 |
| Epe-edaphic | *Dicyrtoma* sp.1 | 0 | 0 | 73±64 | 0 | 20±45 | 7±15 | 7±15 | 0 | 0 | 0 |
| Epe-edaphic | *Dicyrtoma* sp.2 | 0 | 0 | 13±30 | 7±15 | 0 | 7±15 | 33±33 | 0 | 0 | 0 |
| Epe-edaphic | *Dicyrtoma* sp.3 | 0 | 20±30 | 20±45 | 0 | 0 | 0 | 0 | 0 | 0 | 0 |
| Eu-edaphic | *Folsomia* cf. *stella* | 0 | 0 | 0 | 7±15 | 56±77 | 0 | 0 | 0 | 0 | 0 |
| Eu-edaphic | *Folsomia* cf. *villosa* | 280±222 | 0 | 411±409 | 94±89 | 360±353 | 415±336 | 349±364 | 656±334 | 569±393 | 119±110 |
| Eu-edaphic | *Folsomia inoculata* sp.1 | 0 | 0 | 0 | 0 | 0 | 20±45 | 0 | 0 | 0 | 0 |
| Hemi-edaphic | *Folsomia octoculata* sp.1 | 84±188 | 0 | 2267±2737 | 493±868 | 733±1264 | 146±292 | 293±290 | 1368±957 | 405±515 | 0 |
| Hemi-edaphic | *Folsomia octoculata* sp.2 | 308±459 | 288±142 | 4914±5652 | 974±1122 | 1050±1294 | 384±323 | 250±259 | 2348±2320 | 822±385 | 56±77 |
| Eu-edaphic | *Folsomia ozeana* sp. | 28±63 | 0 | 73±86 | 224±216 | 628±832 | 342±233 | 166±71 | 324±495 | 168±183 | 0 |
| Hemi-edaphic | *Folsomia* sp.10 | 0 | 0 | 0 | 0 | 0 | 7±15 | 0 | 0 | 0 | 0 |
| Hemi-edaphic | *Friesea grisea* | 0 | 53±84 | 33±74 | 20±45 | 393±697 | 0 | 53±38 | 33±47 | 0 | 0 |
| Hemi-edaphic | *Friesea laouina* | 0 | 0 | 0 | 13±30 | 0 | 0 | 0 | 0 | 0 | 0 |
| Eu-edaphic | *Heteraphorura seolagensis* | 112±251 | 26±15 | 217±218 | 46±56 | 348±477 | 412±413 | 117±175 | 172±188 | 363±379 | 119±120 |
| Eu-edaphic | *Heteroisotoma sinorossica* | 7±15 | 13±30 | 221±287 | 224±428 | 93±172 | 47±87 | 20±45 | 84±125 | 84±188 | 0 |
| Epe-edaphic | *Homidia similis* | 56±125 | 107±169 | 106±128 | 0 | 7±15 | 13±18 | 0 | 0 | 0 | 0 |
| Epe-edaphic | *Homidia* sp.1 | 0 | 206±144 | 700±853 | 53±65 | 173±189 | 20±30 | 13±30 | 0 | 13±30 | 0 |
| Eu-edaphic | *Hymenaphorura nearctica* | 168±230 | 0 | 28±63 | 168±183 | 54±53 | 66±62 | 387±282 | 216±210 | 209±358 | 63±87 |
| Epe-edaphic | *Isotoma pinnata* | 0 | 0 | 0 | 0 | 0 | 0 | 47±104 | 0 | 13±18 | 0 |
| Hemi-edaphic | *Koreanurina alba* | 0 | 0 | 46±73 | 48±76 | 80±112 | 20±30 | 0 | 0 | 1760±1744 | 0 |
| Eu-edaphic | *Leeonychiurus gulinensis* | 56±125 | 0 | 7±15 | 0 | 0 | 33±41 | 0 | 13±18 | 0 | 0 |
| Epe-edaphic | *Lepidocyrtus* sp.1 | 0 | 0 | 93±64 | 20±45 | 126±116 | 47±65 | 120±150 | 53±119 | 0 | 0 |
| Epe-edaphic | *Lepidocyrtus* sp.2 | 0 | 0 | 353±148 | 233±140 | 0 | 0 | 0 | 0 | 0 | 0 |
| Eu-edaphic | *Lobellina* sp. | 0 | 0 | 28±63 | 0 | 0 | 84±188 | 0 | 0 | 0 | 0 |
| Epe-edaphic | *Metisotoma macnamarai* | 0 | 0 | 20±30 | 0 | 0 | 20±30 | 0 | 0 | 0 | 0 |
| Eu-edaphic | *Micraphorura changbaiensis* | 0 | 0 | 0 | 0 | 0 | 0 | 48±58 | 0 | 0 | 0 |
| Hemi-edaphic | *Morulina* sp. | 0 | 0 | 88±129 | 0 | 0 | 0 | 13±18 | 0 | 0 | 0 |
| Eu-edaphic | *Oligaphorura koreana* | 0 | 7±15 | 0 | 0 | 0 | 7±15 | 40±36 | 20±45 | 0 | 7±15 |
| Epe-edaphic | *Oncopodura yosiiana* | 0 | 0 | 0 | 0 | 106±144 | 0 | 0 | 0 | 0 | 0 |
| Hemi-edaphic | *Pachyotoma sp.1* | 0 | 0 | 0 | 460±1029 | 633±1416 | 553±1182 | 7±15 | 0 | 28±63 | 0 |
| Hemi-edaphic | *Parisotoma* cf. *ekmani* sp.1 | 252±287 | 0 | 189±335 | 93±86 | 86±109 | 60±101 | 177±287 | 27±43 | 583±386 | 252±183 |
| Hemi-edaphic | *Parisotoma* cf. *ekmani* sp.2 | 28±63 | 13±30 | 431±258 | 20±45 | 27±59 | 66±115 | 66±70 | 20±45 | 0 | 0 |
| Hemi-edaphic | *Parisotoma* cf. *hyonosenensis* | 28±63 | 0 | 760±916 | 471±369 | 127±174 | 127±183 | 88±81 | 0 | 425±728 | 0 |
| Epe-edaphic | *Pogonognathellus heterochrous* | 0 | 0 | 40±72 | 7±15 | 0 | 0 | 0 | 0 | 0 | 0 |
| Eu-edaphic | *Protaphorura changbaiensis* | 477±766 | 46±30 | 858±787 | 299±287 | 33±33 | 102±123 | 0 | 0 | 0 | 13±18 |
| Epe-edaphic | *Pseudisotoma sensibilis* sp.1 | 0 | 0 | 7±15 | 0 | 0 | 7±15 | 0 | 0 | 0 | 0 |
| Epe-edaphic | *Pseudisotoma sensibilis* sp.2 | 0 | 0 | 13±30 | 0 | 0 | 0 | 0 | 0 | 0 | 0 |
| Eu-edaphic | *Psyllaphorura raoheensis* | 0 | 0 | 0 | 13±30 | 0 | 7±15 | 60±83 | 0 | 0 | 7±15 |
| Epe-edaphic | *Semicerura* sp.1 | 0 | 0 | 13±18 | 0 | 0 | 0 | 0 | 0 | 0 | 0 |
| Epe-edaphic | *Semicerura draconis* | 0 | 0 | 223±307 | 69±70 | 162±133 | 13±18 | 0 | 0 | 0 | 0 |
| Epe-edaphic | *Semicerura goryshini* | 0 | 0 | 7±15 | 0 | 0 | 28±63 | 0 | 0 | 0 | 0 |
| Eu-edaphic | *Sensillonychiurus pseudoreductus* | 0 | 0 | 20±30 | 0 | 113±126 | 240±518 | 80±96 | 60±134 | 27±59 | 0 |
| Eu-edaphic | *Sensillonychiurus reductus* | 28±63 | 0 | 0 | 60±72 | 80±60 | 46±69 | 94±102 | 20±45 | 28±63 | 0 |
| Eu-edaphic | *Sensillonychiurus virginis* | 56±125 | 13±18 | 146±201 | 33±47 | 40±43 | 113±217 | 26±28 | 0 | 0 | 0 |
| Epe-edaphic | *Sinella* cf. *curviseta* | 0 | 306±487 | 171±256 | 41±62 | 93±128 | 20±30 | 27±59 | 0 | 28±63 | 56±125 |
| Epe-edaphic | *Sinella* cf. *umesaoi* | 0 | 167±209 | 1508±1429 | 435±362 | 133±139 | 128±161 | 7±15 | 0 | 0 | 7±15 |
| Epe-edaphic | *Sminthurinus* sp.1 | 152±286 | 0 | 33±74 | 0 | 0 | 0 | 0 | 0 | 0 | 0 |
| Epe-edaphic | *Sminthurinus* sp.2 | 0 | 0 | 47±51 | 0 | 0 | 0 | 0 | 0 | 0 | 0 |
| Hemi-edaphic | *Subisotoma sp.* | 0 | 0 | 0 | 0 | 0 | 60±89 | 56±125 | 0 | 0 | 0 |
| Hemi-edaphic | *Superodontella* sp.1 | 7±15 | 0 | 54±53 | 27±59 | 87±194 | 86±104 | 153±176 | 60±55 | 13±30 | 200±232 |
| Hemi-edaphic | *Superodontella* sp.2 | 60±116 | 0 | 46±73 | 0 | 13±30 | 0 | 13±30 | 7±15 | 0 | 0 |
| Hemi-edaphic | *Superodontella* sp.3 | 0 | 7±15 | 120±126 | 7±15 | 0 | 0 | 0 | 0 | 20±45 | 140±313 |
| Hemi-edaphic | *Tetracanthella wui* | 0 | 0 | 28±63 | 0 | 474±602 | 1696±1729 | 1023±137 | 0 | 7±15 | 0 |
| Epe-edaphic | *Tomocerina* sp.2 | 0 | 0 | 420±794 | 253±566 | 33±74 | 0 | 0 | 0 | 0 | 0 |
| Epe-edaphic | *Tomocerina varia* | 0 | 0 | 28±63 | 0 | 0 | 60±68 | 53±119 | 0 | 0 | 0 |
| Epe-edaphic | *Tomocerus changbaishanensis* | 0 | 0 | 7±15 | 0 | 0 | 53±119 | 0 | 0 | 0 | 0 |
| Epe-edaphic | *Tomocerus jilinensis* | 0 | 0 | 0 | 7±15 | 0 | 13±18 | 0 | 27±59 | 0 | 0 |
| Epe-edaphic | *Tomocerus kinoshitai* | 0 | 0 | 205±289 | 7±15 | 0 | 0 | 0 | 0 | 28±63 | 0 |
| Epe-edaphic | *Tomocerus laxalamella* | 0 | 0 | 60±134 | 0 | 0 | 0 | 0 | 0 | 0 | 0 |
| Epe-edaphic | *Tomocerus nigrus* | 0 | 0 | 0 | 0 | 0 | 0 | 7±15 | 0 | 0 | 0 |
| Epe-edaphic | *Tomocerus* sp.1 | 0 | 193±379 | 176±177 | 33±47 | 40±72 | 0 | 0 | 20±45 | 84±188 | 0 |
| Epe-edaphic | *Tomocerus* sp.2 | 28±63 | 0 | 0 | 0 | 0 | 0 | 0 | 0 | 0 | 0 |
| Epe-edaphic | *Vertagopus asiaticus* | 0 | 0 | 0 | 0 | 13±30 | 0 | 0 | 0 | 0 | 0 |
| Epe-edaphic | *Tomocerina* sp.1 | 0 | 0 | 80±107 | 120±117 | 27±59 | 0 | 46±73 | 40±89 | 0 | 0 |
